# Investigating the Impact of Vitamin A and Amino Acids on Immune Responses in Celiac Disease Patients

**DOI:** 10.3390/diseases12010013

**Published:** 2024-01-01

**Authors:** Shayan Fallah, Nastaran Asri, Abdolrahim Nikzamir, Shokoufeh Ahmadipour, Amir Sadeghi, Kamran Rostami, Mohammad Rostami-Nejad

**Affiliations:** 1 Department of Clinical Biochemistry, School of Medicine, Shahid Beheshti University of Medical Sciences, Tehran 1985717411, Iran; shayan_flh96@yahoo.com (S.F.); nikzamirar@yahoo.com (A.N.); 2 Celiac Disease and Gluten Related Disorders Research Center, Research Institute for Gastroenterology and Liver Diseases, Shahid Beheshti University of Medical Sciences, Tehran 1985717411, Iran; nastaran.asri26@gmail.com; 3 Department of Pediatric, Faculty of Medicine, Lorestan University of Medical Sciences, Khorramabad 6813833946, Iran; shokoufeh.ahmadipour@gmail.com; 4 Gastroenterology and Liver Diseases Research Center, Research Institute for Gastroenterology and Liver Diseases, Shahid Beheshti University of Medical Sciences, Tehran 1985717411, Iran; amirsadeghimd188@gmail.com; 5 Department of Gastroenterology, MidCentral DHB, Palmerston North 4442, New Zealand

**Keywords:** celiac disease, gluten free diet, amino acids, vitamin A, treatment

## Abstract

Amino acids (AAs) and vitamin imbalances are observed in celiac disease (CD). This study evaluated the plasma profile of vitamin A and AAs and the expression level of IL-2, IL-4, IL-10, IL-12 and TGFβ in CD patients. A total of 60 children and adults with CD and 40 healthy controls (HCs) were included. The plasma profile of Vitamin A and AAs and the mRNA expression levels of target genes were assessed. Active adult patients exhibited a decrease in Vitamin A levels (*p* = 0.04) and an increase in IL-2 (*p* = 0.008) and IL-12 (*p* = 0.007) mRNA expression compared to the HCs. The treated adult patients showed elevated Serine (*p* = 0.003) and Glycine (*p* = 0.04) levels, as well as increased IL-12 (*p* < 0.0001) mRNA expression, and a decrease in Tryptophan (*p* = 0.04) levels relative to the controls. Additionally, the treated adult patients had higher plasma levels of Threonine compared to both the active (*p* = 0.04) and control (*p* = 0.02) subjects, and the increased mRNA expression of IL-4 (*p* = 0.01) in comparison to the active patients. In active children with CD, the IL-2 mRNA level was found to be higher than in the controls (*p* < 0.0001) and in the treated children (*p* = 0.005). The treated children with CD exhibited decreased plasma levels of Tryptophan (*p* = 0.01) and Isoleucine (*p* = 0.01) relative to the controls, and the increased mRNA expression of TGFβ (*p* = 0.04) relative to the active patients. Elevated levels of specific AAs (Serine, Glycine, Threonine) in the treated CD patients suggested their potential to improve intestinal damage and inflammation, while decreased levels of Tryptophan and Isoleucine highlighted the need for dietary intervention.

## 1. Introduction

Celiac disease (CD) is a chronic intestinal inflammatory disorder caused by an intolerance to gluten protein in genetically susceptible individuals [1]. According to the prevailing consensus, the prominent predisposing factors for the development of CD are human leukocyte antigen (HLA)-DQ2 and HLA-DQ8 [2]. This disorder can present with intestinal (bloating, chronic diarrhea, dyspepsia) and extra-intestinal (chronic anemia, fatigue, osteoporosis, infertility) symptoms, and some CD patients do not exhibit any of the classical manifestations [3]. Abnormal T-cell-mediated immune responses to dietary gluten lead to the massive production of pro-inflammatory cytokines [4]. There are also endogenous immunoregulatory mechanisms, although not sufficient, that involve negative feedback mechanisms; these include the secretion of anti-inflammatory cytokines, which attempt to counterbalance these abnormal immune responses [5]. The inflammation caused by immune responses to ingested gluten leads to villous atrophy and crypt hyperplasia in the small intestine [6]. Villous atrophy is accompanied by a decrease in the surface area that is available for nutrient absorption in the small bowel [7]. Adherence to a lifelong gluten-free diet (GFD) is considered to be the only available treatment for CD patients [8]. Gluten-containing grains are an essential part of a healthy diet and are considered a good source of complex carbohydrates, some important vitamins, minerals and amino acids (AAs); eliminating them from the diet may cause health problems [9,10]. Therefore, monitoring the changes in the essential micronutrient levels that occur during CD pathogenesis and assessing the impact of GFD on these changes is of great importance [11,12]. Among the essential micronutrients, vitamin A and amino acids play important roles in regulating immune responses and maintaining intestinal homeostasis. Retinoic acid (RA), the vitamin A metabolite, affects both adaptive and innate immune responses and plays an important role in inducing effector CD4+ T cell responses during infection [13,14]. RA, through binding to its receptors, can drive Th1/Th2 differentiation towards Th2, induce the TGF-β-dependent conversion of naïve T cells into Foxp3+ regulatory T cells, and mediate immune homeostasis [14,15,16,17]. It can also affect the expression of both pro-inflammatory (like IFN-γ and IL-12) and anti-inflammatory (like IL-10 and IL-4) production [17,18]. Recent studies have indicated that AAs play a crucial role in regulating inflammatory responses and can modulate the expression of pro- and anti-inflammatory cytokines [9,19]. Indoleamine 2,3-dioxygenase (IDO), as an enzyme with high expression in the intestinal biopsy samples of CD patients, can metabolize tryptophan (TRP) to kynurenine, which has anti-inflammatory properties [20,21,22]. Moreover, increased histidine (HIS), glycine (GLY) and arginine (ARG) levels could enhance the vulnerability of potential CD patients to intestinal inflammation [23]. The roles of methionine (MET), threonine (THR), His, and several other AAs in improving intestinal villus morphology, intestinal barrier integrity and regulating immune responses have also been demonstrated [24]. Additionally, ameliorating gut inflammation via serine (SER), glutamine (GLN) and glutamate (GLU) has been discussed [25,26,27]. In the present study, we evaluated the changes in the plasma levels of vitamin A and AAs in adults and children patients with CD relative to the healthy controls, and evaluated the effects of these changes on the expression level of IL-2, IL-4, IL-10, IL-12 and TGFβ.

## 2. Materials and Methods

### 2.1. Recruitment of Participants and Peripheral Blood Sample Collection

We studied 30 newly diagnosed (active) CD patients: 15 children (mean age 9.20 ± 3.27 years) and 15 adults (mean age 31.8 ± 12.71 years); and 30 treated CD subjects (by adhering to a strict GFD): 15 children (mean age 10.60 ± 2.92 years) and 15 adults (mean age 39.0 ± 8.87 years). CD patients were recruited between February 2021 and June 2022 from the Celiac Disease and Gluten-Related Disorders Research Center, Shahid Beheshti University of Medical Sciences. The inclusion criteria were confirmed CD according to the European Society for Pediatric Gastroenterology Hepatology and Nutrition (ESPGHAN) criteria in children [28] and according to the American Gastroenterological Association (AGA) recommendations in adults [29]. Moreover, 40 healthy subjects, including 20 children (mean age 10.9 ± 3.97 years) and 20 adults (mean age 35.25 ± 10.7 years) with no clinical and serological evidence of CD or any other immune-related diseases up to their first-degree who were willing to participate in the study, were recruited as a control group. Pregnant and lactating women, subjects with other autoimmune and inflammatory conditions, acute or chronic diseases, cancer, or gastrointestinal infections were excluded from the study.

Venous peripheral blood samples (10 mL) were carefully obtained from the study participants between 8:00 and 8:30 a.m. after an overnight fasting of at least 12 h. All samples were subjected to identical experimental conditions, like transfer temperature, for consistent observations. Demographic data, clinical symptoms, and self-reported dietary habits were recorded earlier than the blood sampling. In fact, the self-reported amount of meat, fish, eggs, pulses, dairy products, fats, vegetables, fruits, sweets, and nuts consumed in the 1 month prior to the study was acquired with a questionnaire and categorized as sufficient or insufficient (according to the Asian food pyramid) [30].

The study was approved by the ethical committee of the Research Institute for Gastroenterology and Liver Diseases (RIGLD), Shahid Beheshti University of Medical Sciences, Tehran, Iran (IR.SBMU.MSP. REC.1397.564), and written informed consent was obtained from all included subjects before participation.

### 2.2. Metabolite Analysis

Whole blood samples were centrifuged at 3500× *g* for 15 min, and the resulting plasma fraction was immediately stored at −80 °C until used for the HPLC analysis of the Vitamin A and total amino acids profiles. An HPLC series system (ACME 9000 system, Younglin, Anyang, Korea) with a UV detector (for Vitamin A levels) and a fluorescence detector (for amino acids levels) was used in the present study. Plasma samples were deproteinized by adding a methanolic solution [31]. After vortexing for 30 sec and centrifugation for 7 min at 5000× *g*, the clear supernatant was used for further analysis. For Vitamin A analysis, 100 λ of the supernatant was injected into the C18 column (250 mm × 4.6 mm, 5 µm) along with methanol–ethanol (3:1 v/v) as the mobile phase, and the UV signals were recorded at 295 nm. Moreover, the chromatographic separation of AAs was achieved by adding 100 λ of the clear supernatant to the GL Sciences column (250 mm × 3.0 mm, 3 μm) using methanol–Tetrahydrofuran (4:1 v/v) as the mobile phase, and the fluorescence signals were recorded at the optimal excitation and emission wavelength (ex: 340 nm, em: 450 nm).

### 2.3. RNA Extraction and cDNA Synthesis

Total ribonucleic acid (RNA) was isolated from the whole blood samples of all participants using a YTA Total RNA Purification Mini kit for Blood/Cultured Cell/Tissue (Yekta Tajhiz Azma, Tehran, Iran) according to the manufacturer’s instructions. The RNA concentration and quality were evaluated using a NanoDrop 1000 spectrophotometer (NanoDrop Fisher Thermo, Wilmington, DE, USA). After adjusting the RNA concentrations, cDNA synthesis was performed using the 2 Step 2X RTPCR Premix (Taq) kit (BioFact™, Daejeon, Korea), and the cDNA samples were stored at −20 °C for quantitative real-time PCR.

### 2.4. Primer Designing and Quantitative Real-Time PCR (RT-qPCR)

The specific primers used for the amplification of IL-2, IL-4, IL-10, IL-12, TGFβ and Beta- 2-microglobulin (B2M), as a housekeeping gene, were designed using the Gene Runner (version 3.05) software. These sequences were analyzed using PrimerBlast in the NCBI database (http://blast.ncbi.nlm.Nih.gov/ (accessed on 1 May 2022)), and their properties are shown in Supplementary Table S1. The mRNA expression levels of the target genes were assessed using the SYBR Premix Ex Taq (RealQ Plus 2x Master Mix Green-Amplicon, Japan using Rotor-Gene^®^ Q (Qiagen, Germany) real-time PCR system. All qPCR reactions were performed in duplicates and the mRNA expression level of each gene was calculated according to delta-Ct (ΔCt = ΔCt target − ΔCt endogenous), and was presented in graphs using the comparative Ct method (2^−ΔΔCt^).

### 2.5. Statistical Analysis

Data analysis was conducted utilizing Statistical Package for the Social Sciences (SPSS) version 25.0, developed by SPSS Inc. (Chicago, IL, USA). Graphical representations were created using GRAPHPAD Prism 8.4.0, a software product by GraphPad Software, Inc. (San Diego, CA, USA). The data were expressed as the mean ± standard deviation (SD). To evaluate the variations across groups, one-way analysis of variance (ANOVA) was employed. The correlation between variables was assessed using Pearson’s correlation tests. A significance level of *p*-value < 0.05 was considered statistically significant.

## 3. Results

### 3.1. Demographic and Clinical Characteristics

The demographic characteristics of the study subjects are reported in Table 1. Briefly, 60 confirmed CD patients, including 30 treated subjects (15 children and 15 adults) and 30 active patients (15 children and 15 adults), and 40 healthy controls were included. The case and control participants in both the pediatric and adult groups were matched for age, BMI and gender, and there was no significant difference in this regard between the studied groups (*p* > 0.05) (Table 1). 

The HLA-DQ2 haplotype was observed in 60% of active CD adults, in 80% of treated CD adults, in 80% of active CD children, and in 73.3% of treated CD children (Supplementary Figure S1). According to the Marsh classification, most of the patients in the treated adult (80%), active adult (80%), and treated pediatric groups (40%) were in the Marsh III stage (at the time of diagnosis) (Supplementary Figures S1 and S2). 

**Figure 1:**
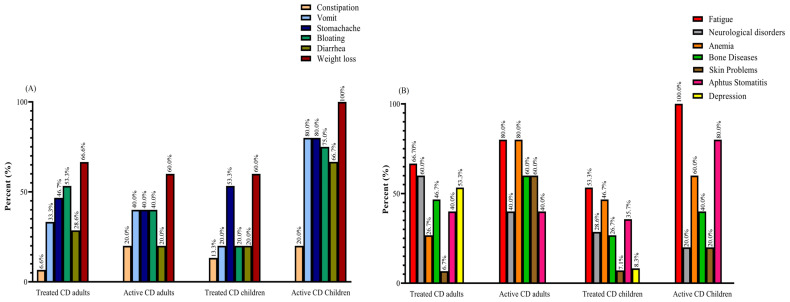
(**A**) GI and (**B**) non-GI symptoms reported by active and treated CD patients: most patients complained of two or more symptoms. Abbreviations: CD: Celiac disease; GI: Gastrointestinal.

Weight loss was reported as the most prevalent gastrointestinal symptom among all groups of patients with CD, with rates of 60% in active and treated adults and treated pediatrics, and 100% in active pediatrics. Meanwhile, fatigue emerged as the most common non-gastrointestinal symptom, with percentages of 66.7% in treated adults, 80% in active adults, 53.3% in treated children, and 100% in active children. Among the active CD adults, both fatigue and anemia were the most frequently reported symptoms, with rates of 80% (Figure 1).

### 3.2. Vitamin A Levels

**Figure 2:**
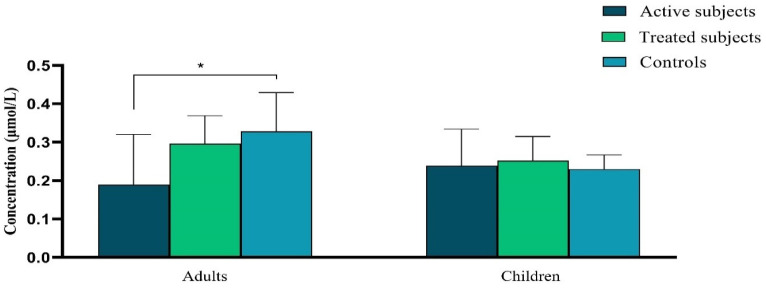
The difference in plasma vitamin A levels (μmol/L) between active and treated CD patients and controls. Abbreviation: CD: Celiac disease. *: *p* ≤ 0.05.

Our data demonstrated a significant decrease in the plasma level of Vitamin A in active CD adult patients in comparison to the control adults (*p* = 0.04). There was not any significant difference in the plasma level of Vitamin A between pediatric groups (*p* ˃ 0.05) (Figure 2).

### 3.3. Amino Acids Profiles

**Figure 3:**
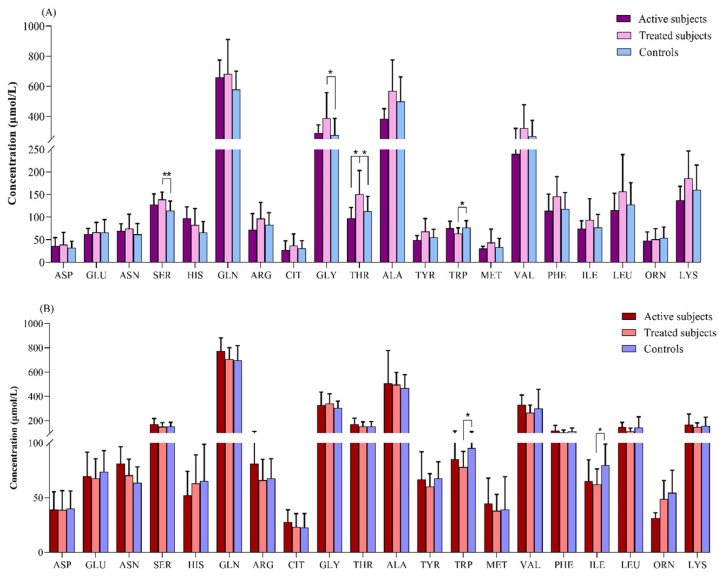
Comparison of amino acids levels (μmol/L) between (**A**) adult and (**B**) pediatric patients with CD relative to the controls. *: *p* ≤ 0.05, **: *p* ≤ 0.01. Abbreviations: ALA: Alanine; ARG: Arginine; ASN: Asparagine; ASP: Aspartate; CD: Celiac disease; CIT: Citrulline; GLN: Glutamine; GLU: Glutamate; GLY: Glycine; HIS: Histidine; ILE: Isoleucine; LEU: Leucine; LYS: Lysine; MET: Methionine; ORN: Ornithine; PHE: Phenylalanine; SER: Serine; THR: Threonine; TRP: Tryptophan; TYR: Tyrosine; VAL: Valine.

Our results revealed that in the comparison between the adult groups, the plasma levels of SER (*p* = 0.003) and GLY (*p* = 0.04) were significantly higher in the treated CD patients than in the controls. The THR level was also increased in the treated CD patients compared to the active CD (*p* = 0.04) and control (*p* = 0.02) subjects. In contrast, the treated CD patients showed a lower concentration of TRP than the controls (*p* = 0.04) (Figure 3A).

The TRP and ILE levels were found to be significantly lower in the treated pediatric CD patients relative to the controls (*p* = 0.01 for both of them) (Figure 3B).

### 3.4. mRNA Expression Analysis

**Figure 4:**
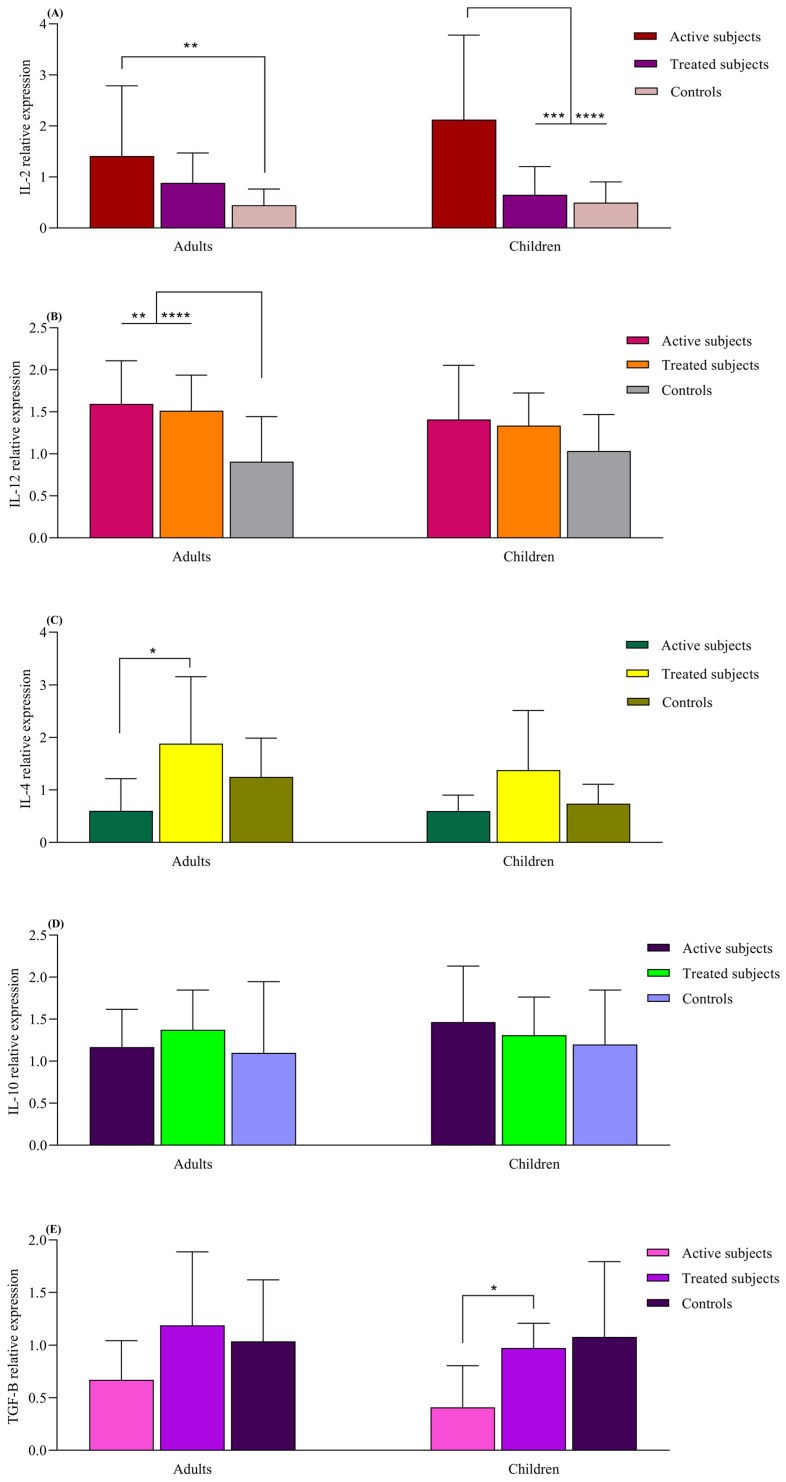
Analysis of relative expression levels in CD patients compared to controls using real-time PCR assay. All expression levels are normalized to that of B2M. The analyzed genes were as follows: (**A**) IL-2, (**B**) IL-12, (**C**) IL-4, (**D**) IL-10 and (**E**) TGF-β. Data are presented as mean  ±  S.D. *: *p* ≤ 0.05, **: *p* ≤ 0.01, ***: *p* ≤ 0.001, ****: *p* ≤ 0.0001. Abbreviations: B2M: Beta- 2-microglobulin; CD: Celiac disease: IL: Interleukin; TGF-β: Transforming growth factor-β.

The IL-2 mRNA expression was higher in the active adult CD patients compared to the healthy adult controls (*p* = 0.008). The active CD children also exhibited higher IL-2 expression compared to the treated (*p* = 0.005) and control children groups (*p* < 0.0001). The adult CD patients, both active and treated, displayed higher IL-12 mRNA levels than the adult control subjects (*p* = 0.007 and *p* < 0.0001, respectively). Although this pattern was also observed in pediatric groups, the differences were not statistically significant (*p* > 0.05). IL-4 mRNA expression was increased in the adult treated CD patients compared to those with active CD (*p* = 0.01). The IL-4 expression in pediatric groups resembled that of the adult groups, but no significant difference was found (*p* > 0.05). Children with treated CD had higher TGFβ expression than those with the active form of the disease (*p* = 0.04). Similar observations were made in adult groups, but the difference was not found to be statistically significant (*p* > 0.05). The levels of IL-10 did not significantly differ between the children and adult groups (*p* > 0.05) (Figure 4).

### 3.5. Dietary Habits

According to the data, the adults with active CD had a reduced intake of fish. Similarly, both the treated adults and children had a considerably lower consumption of dairy products.

### 3.6. Correlation Analysis

**Figure 5:**
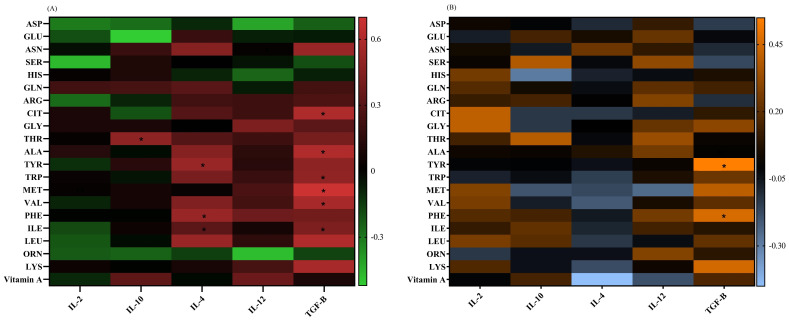
A heat map of the correlations between the (**A**) vitamin A and amino acid levels and gene expressions in adults, red: positive correlations; green: negative correlations; and (**B**) vitamin A and amino acid levels and gene expressions in pediatric patients, orange: positive correlations; blue: negative correlations. Significant correlations (*p* ˂ 0.05) are marked with a black asterisk. *: *p* < 0.05.

The IL-4 mRNA level had positive correlations with the ILE (*p* = 0.04, r = 0.31), PHE (*p* = 0.02, r = 0.52), and TYR (*p* = 0.02, r = 0.52) concentrations in adults. The TGF-β mRNA expression showed positive correlations with ILE (*p* = 0.04, r = 0.43), VAL (*p* = 0.04, r = 0.57), MET (*p* = 0.007, r = 0.7), TRP (*p* = 0.04, r = 0.49), ALA (*p* = 0.03, r = 0.6), and CIT (*p* = 0.03, r = 0.59) in adults, as well as with PHE (*p* = 0.04, r = 0.46) and TYR (*p* = 0.01, r = 0.55) in children. The IL-10 mRNA levels had a positive correlation with the THR (*p* = 0.03, r = 0.48) plasma levels in adults (Figure 5).

## 4. Discussion

Our results showed that the adult patients with CD had significantly lower levels of plasma vitamin A compared to the adult controls, but there was not any significant difference between pediatrics in this regard. This finding is consistent with previous reports. For instance, Wierdsma et al. evaluated the nutritional and vitamin/mineral status of early diagnosed adult CD patients when compared to healthy individuals. According to their results, 7.5% of patients had a deficiency of vitamin A [10]. Weintraub et al., in a study on active children with CD, did not observe an association between CD and vitamin A deficiency [32]. It should be noted that the lower quantity of fish consumed by our participating adults with active CD may have had a negative effect on their vitamin A levels. The lack of difference observed in the vitamin A levels between children with CD and the controls could be attributed to the close monitoring that pediatric patients often receive compared to adults. Additionally, adults with CD may have experienced the condition for a longer time, leading to more extensive damage to the small intestine. This increased damage could result in a greater impairment of nutrient absorption, including vitamin A. Due to the important role that RA plays, as a vitamin A metabolite, in alleviating intestinal inflammation and affecting adaptive and innate immune responses, controlling its level in CD patients is of great importance [13,14]. 

Through the examination of plasma AA levels, we observed significantly elevated levels of SER and GLY in the treated adult patients with CD compared to the control subjects. Moreover, the level of THR was found to be higher in the CD patients receiving treatment in comparison to both the active CD patients and the individuals in the control group. SER, GLY, and THR are known as AAs involved in maintaining intestinal barrier integrity and possess anti-inflammatory properties [33,34,35]. The elevation of these Aas in treated patients may contribute to the improvement of intestinal damage. Crucially, THR plays a significant role in the composition of intestinal mucins and IgA. These substances are produced in large quantities during inflammation, aiding in the restoration of intestinal balance and regulating the body’s pro- and anti-inflammatory reactions [36,37,38]. In the current study, the THR concentration showed a positive correlation with the IL-10 mRNA level, a cytokine that plays a critical role in preventing inflammatory responses. This may suggest that THR could potentially be used as a therapeutic option for treating CD [39].

Importantly, the plasma concentration of TRP was reduced in our treated CD adults and pediatrics. A limited intake of TRP could potentially impede the process of intestinal healing, as TRP catabolism serves as a crucial factor in the regulation of intestinal inflammation [22]. According to reports, a high TRP diet increases the production of aryl hydrocarbon receptor (AhR) ligands and activates this pathway, which is responsible for controlling inflammation and safeguarding the gut barrier; it also decreases gluten immunopathology in mice expressing the DQ8 gene [40,41]. As a result, there is a hypothesis suggesting that supplementing with TRP could potentially serve as a new treatment for CD patients. TRP has also been shown to promote the production of TGF-β cytokines and activate the TGF-β signaling pathway [42]. In the current study, a positive correlation was observed between TGF-β and TRP. Pediatrics with GFD-treated CD had an increased expression of TGFβ compared to the active subjects. This change was also observed in the adult groups but was insignificant. Since TGFβ is an anti-inflammatory cytokine with a regulatory effect on immune homeostasis, the impairment of TGFβ signaling may be associated with intestinal inflammation [43] and the decrease in TGFβ gene expression observed in active CD subjects is justified. The level of TGF-β showed a positive correlation with ILE, VAL, MET, ALA, PHE, TYR and CIT too.

The ILE level was also significantly lower in the treated pediatric CD patients relative to the controls. ILE is a crucial amino acid acknowledged for its role in controlling immune function, particularly in the production of substances important for immune response [44]. In fact, the administration of l-isoleucine can effectively manage and treat colitis induced by dextran sulfate sodium (DSS) in rats [44,45]. A notable outcome of this treatment is its ability to boost the levels of IL-4 in the colon of rats [44]. In the current study, the treated adults with CD had higher levels of IL-4 mRNA than the active CD adult subjects, and there was a significant positive correlation between the level of this cytokine and the ILE level. Due to the potential protective role of IL4 toward the inflammatory processes occurring in the gut mucosa of CD patients, evaluating the potential of ILE to be used as another amino acid supplement for alleviating CD patients’ inflammation is of great importance. Furthermore, the presence of IL-4 had been shown to negatively impact the activity of IDO, an enzyme responsible for breaking down tryptophan [46]. Therefore, the increased levels of IL-4 observed in the adults with CD who were treated with a GFD could be attributed to the prevention of total TRP loss in this group (as previously mentioned, the level of TRP had decreased in the GFD-treated adult CD patients). On the other hand, since vitamin A is a positive regulator of IL-4, the low level of this cytokine in active CD adults may be related to the low level of vitamin A in this group [47]. This cytokine also showed positive correlations with PHE and TYR. 

IL-2 mRNA expression was higher in our studied adult patients with active CD than in the healthy adult controls, and higher in the children with active CD than in the treated and control children groups. In this regard, Manavalan et al. demonstrated a significant elevation in IL-2 levels in active CD patients relative to the controls and also in patients who were on GFD for less than 1 year; this is in comparison to the patients on GFD for more than 1 year. In fact, IL-2 is among the important mediators of the Th-1 immune response, and its role in CD is well documented [48]. Its level increases after one-off gluten ingestion and it is considered to be a potential diagnostic biomarker for CD [49,50]. The active and treated adult CD patients also had higher IL-12 mRNA levels in comparison to the control subjects. Since IL-12 is effective in the expansion of naive CD4+ T cells to Th1 cells, which are the important cells in CD pathogenesis, the increase in this cytokine in the active CD groups was not unexpected [51]. On the other hand, the regulatory effects of this cytokine on immune responses and its positive effect on the expansion and increase in regulatory T cells have also been reported in studies that justify its increase in the treated groups [52]. According to Björck et al., the serum level of IL-12 is significantly increased in children with CD and decreases following GFD adherence [53]. The mucosal increase in IL-12 during intestinal inflammation has also been observed [54]. It has been shown that SER, HIS and TYR are required for IL-2 binding and biological activity, and that ASP, THR, and TRP are among the important amino acids for the interaction of IL-12 with its receptor [55]. Therefore, controlling these residues may have the potential to be considered a therapeutic target for CD patients, but this needs to be confirmed in further studies [56,57].

## 5. Conclusions

In summary, the elevation of certain amino acids, such as SER, GLY, and THR, in treated adult CD patients suggests their potential role in improving intestinal damage and inflammation. On the other hand, decreased levels of TRP and ILE in treated CD patients indicate the need for dietary intervention to ensure the adequate intake of these essential amino acids. The positive correlations observed between certain cytokines (IL-10, IL-4) and amino acids (THR, ILE) further support their potential as therapeutic targets in CD treatment. Further research is warranted to explore the clinical implications of these findings and validate the use of amino acids as biomarkers and targets for CD management.

## Figures and Tables

**Table 1:** Demographic characteristics of study groups.

**Adults**					
Variables Groups	Number	Gender		Age	BMI
		Female	Male		
Controls	20	10 (50%)	10 (50%)	35.25 ± 10.7	22.06 ± 8.33
Treated	15	8 (53.3%)	7 (46.7%)	39.0 ± 8.87	26.28 ± 4.57
Active	15	10 (66.6%)	5 (33.3%)	31.8 ± 12.71	21.68 ± 4.54
*p*-value		0.49		0.34	0.12
**Children**					
Variables Groups	Number	Gender		Age	BMI
		Girl	Boy		
Controls	20	10 (50%)	10 (50%)	10.9 ± 3.97	19.69 ± 4.45
Treated	15	8 (53.3%)	7 (46.7%)	10.60 ± 2.92	13.77 ± 12.63
Active	15	9 (60%)	6 (40%)	9.20 ± 3.27	14.75 ± 3.81
*p*-value		0.92		0.63	0.16

## Data Availability

The data presented in this study are available upon request from the corresponding author.

## Supplementary Materials

The following supporting information can be downloaded at: https://www.mdpi.com/article/10.3390/diseases12010013/s1, Figure S1: HLA status of studied patients; Figure S2. Histological classification of studied patients; Table S1: Sequence of primers used in real-time PCR.
