# Supplementary material for: Investigating the Impact of Vitamin A and Amino Acids on Immune Responses in Celiac Disease Patients
Source: Diseases. 2024 Jan 1;12(1):13. doi: 10.3390/diseases12010013 (PMC10814138; doi:10.3390/diseases12010013)
Supplement: Supplementary file 1 [file diseases-12-00013-s001.zip › diseases-2632688-supplementary.pdf]

Supplementary tables

Table S1. Sequence of primers used in real-time PCR

Gene Symbol	**Forward primer**	**Reverse primer**
IL-2	5'-TACATGCCCAAGAAGGCCAC-3'	5'-AGCACTTCCTCCAGAGGTTTG-3'
IL-4	5'-CTTTGCTGCCTCCAAGAACAC-3'	5'-TTCCTGTCGAGCCGTTTCAG-3'
IL-10	5'- AAGAAGGCATGCACAGCTCA -3'	5'- AAGTGGGTGCAGCTGTTCTC -3
IL-12	5'-GTGAGGTCTTAGGCTCTGGC-3'	5'-AACCTCGCCTCCTTTGTGAC-3'
TGFβ	5'-CAATTCCTGGCGATACCTCAG-3'	5'-GCACAACTCCGGTGACATCAA-3'
B2M	5'-CCAGCGTACTCCAAAGATTC-3'	5'-ATGTCGGATGGATGAAACCC-3'

Supplementary Figures

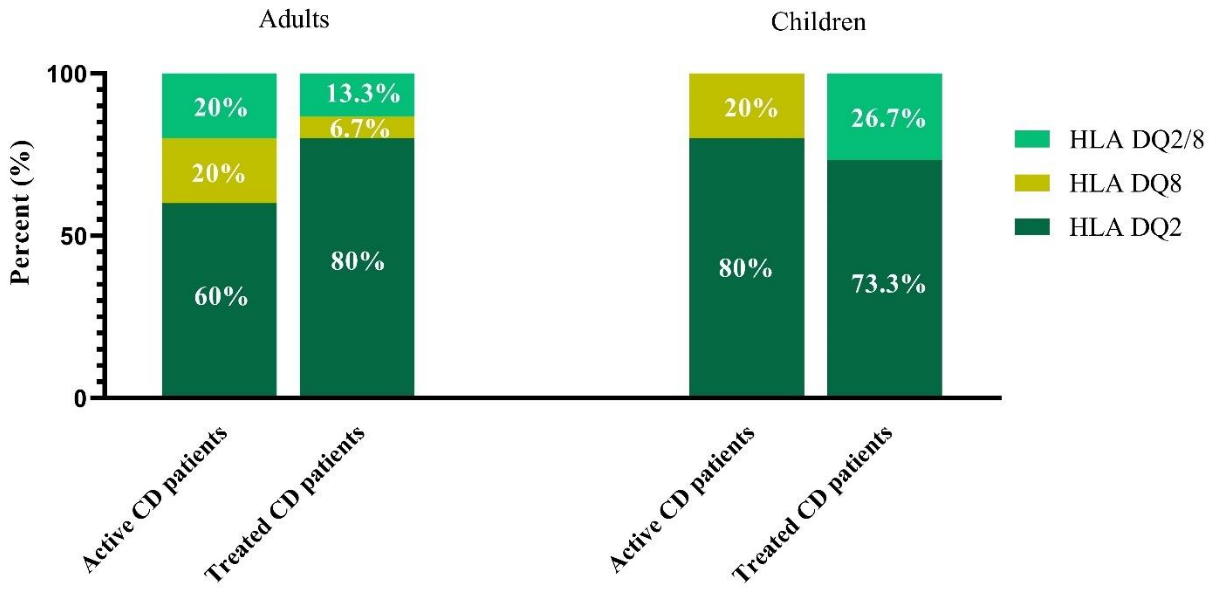

Figure S1. HLA status of studied patients

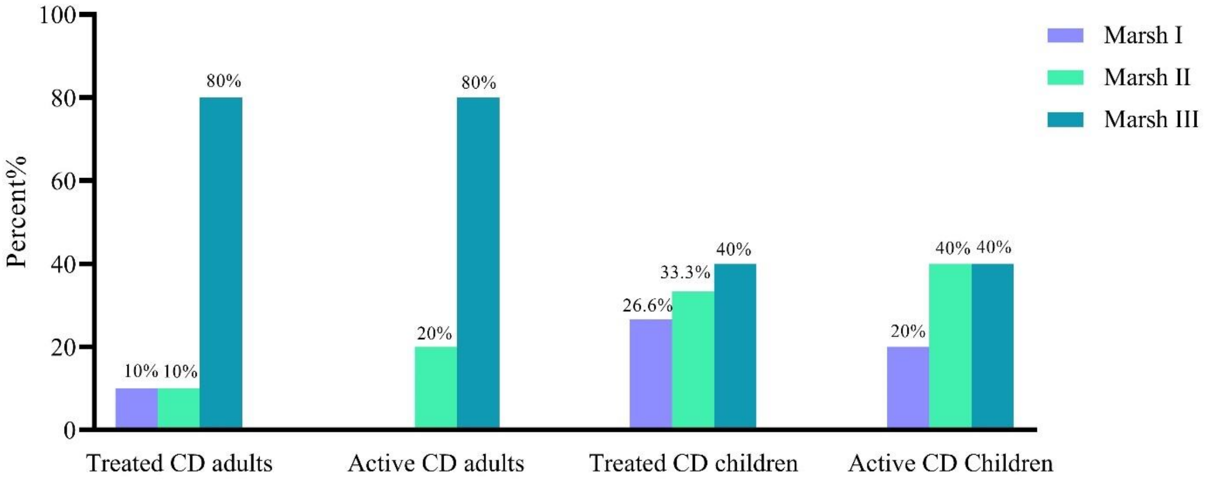

Figure S2. Histological classification of studied patients
